# Comparative Kinetics of Supported Lipid Bilayer Formation on Silica Coated Vertically Oriented Highly Curved Nanowires and Planar Silica Surfaces

**DOI:** 10.1021/acs.nanolett.4c05303

**Published:** 2025-02-06

**Authors:** Julia Valderas-Gutiérrez, Rubina Davtyan, Christelle N. Prinz, Emma Sparr, Peter Jönsson, Heiner Linke, Fredrik Höök

**Affiliations:** † NanoLund, Lund University, P.O. Box 118, SE-22100 Lund, Sweden; ‡ Solid State Physics, Lund University, P.O. Box 118, SE-22100 Lund, Sweden; § Physical Chemistry, Lund University, P.O. Box 124, SE-22100 Lund, Sweden; ∥ Department of Physics, Chalmers University of Technology, SE-41296 Göteborg, Sweden

**Keywords:** supported lipid bilayer, semiconductor nanowires, lightguiding, signal enhancement, epifluorescence microscopy

## Abstract

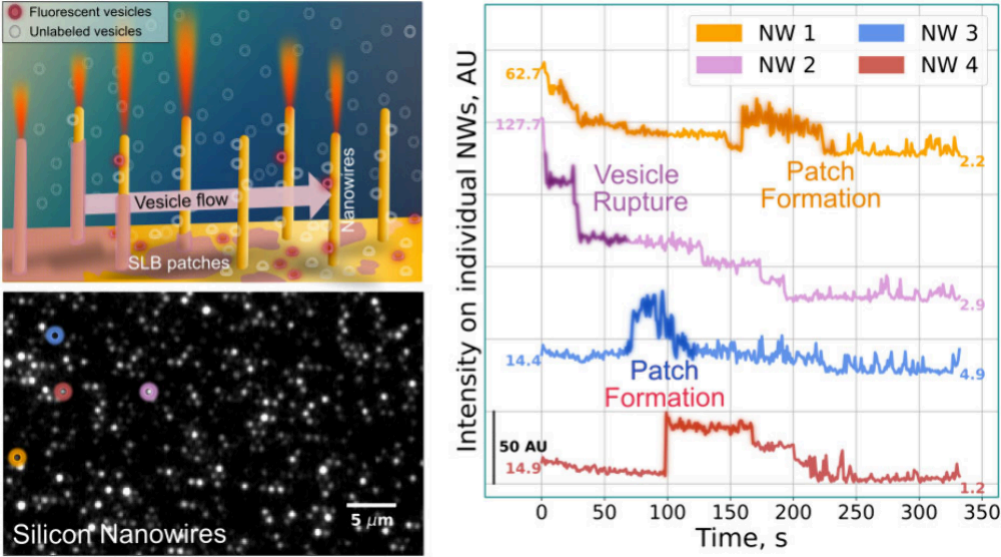

Supported lipid bilayers (SLBs), formed via lipid vesicle adsorption on highly curved silica surfaces, are widely used in biosensor applications and as models for curved cell membranes. However, SLB formation is often hindered on convex structures with radii comparable to the vesicles. In this study, lightguiding semiconductor nanowires (NWs), engineered for fluorescence signal enhancement, were used to compare the kinetics of SLB formation on vertically oriented NWs and planar silica surfaces. Time resolved fluorescence microscopy with single-molecule sensitivity revealed that while vesicle adsorption rates were similar on both surfaces lateral expansion of the SLB was up to three times faster on NWs than on the planar control. This accelerated expansion is attributed to lower energy penalties when SLBs spread along the cylindrical NWs compared with a planar surface, accompanied by accelerated SLB expansion driven by the merging of the SLB with excess lipids from vesicles accumulated on the NWs.

Despite the complexity of the cellular membranes that compartmentalize living cells and their internal organelles, simplified models have provided crucial insights into the molecular self-assembly and physicochemical properties of lipid membranes and their associated proteins.^1,2^ Supported lipid bilayers (SLBs) serve as one of the most attractive mimics of the cell membrane,^1,2^ particularly due to their compatibility with a wide range of surface analytical tools, including label free ensemble averaging,^3−7^ as well as techniques offering single lipid vesicle and biomolecule resolution, such as atomic force microscopy (AFM),^8,9^ total internal reflection fluorescence (TIRF) microscopy^10,11^ and label-free scattering imaging.^12,13^

A commonly used method for forming SLBs utilizes direct lipid vesicle adsorption. On primarily silica-based substrates, surface-induced vesicle collapse into small-scale SLB patches is initiated when a critical vesicle coverage is reached, followed by spontaneous formation of planar, continuous and laterally fluid SLBs.^14,15^ Investigations of SLBs of varying molecular complexity have provided detailed insights into several aspects of relevance for cell membrane biology, including cellular signaling,^16^ receptor–ligand and protein interactions,^17^ viral interactions,^18^ and immune synapse formation and activation.^2,19^ Recent efforts have also combined SLBs with nanostructured surfaces to study the effect of membrane curvature on biomolecular binding, self-assembly processes and lipid domain formation,^20−22^ as well as for bioanalytic sensing applications.^23^

In the realm of biocompatible functionalization for nanoscale sensor elements, SLBs have been integrated with sophisticated nanosculptured substrates, such as fluorescent nanodiamonds,^24^ nanoparticles,^25−27^ as well as suspended nanowires.^28,29^ Notably, arrays of vertical nanowires (NWs) have also gained attention in this field.^30^ While traditionally employed in electronics and photovoltaic energy applications,^31^ semiconductor NWs have more recently emerged as versatile platforms for fluorescence-enhanced imaging and biosensing,^32−35^ as well as for cell probing and manipulation.^36,37^ Spontaneous formation of fluid SLBs from zwitterionic lipid vesicles on silica-coated gallium phosphide nanowires has also been demonstrated,^38^ providing platforms for protein anchoring while preserving high mobility.^27,38,39^

While supported lipid bilayers (SLBs) have been observed to form spontaneously on surfaces with high curvature,^27,38,39^ literature reports also suggest that curvature can impede the SLB formation process.^40,41^ For instance, the influence of curvature becomes more pronounced as the radius of curvature (ROC) of convex nanostructures approaches the dimensions of vesicles,^42^ while SLB formation is favored in concave regions.^43^ Given these contrasting observations on the effect of curvature on the SLB formation process, vertically oriented high-refractive index NWs combine two properties that make them well-suited for investigating the impact of surface curvature on the SLB formation process. Their ROC can be matched to that of lipid vesicles while simultaneously supporting optical waveguide modes, enhancing the excitation and emission of surface-bound fluorophores while focusing the emission directionally at the tip of the NWs, which significantly improves the signal-to-noise ratio,^34^ enabling single-molecule sensitivity using conventional microscopy setups.^39^

Here, we utilized these unique features of vertically oriented lightguiding NWs to investigate the kinetics of SLB formation using lipid vesicles of similar diameter to that of the NWs and compared the results to SLB formation on planar silica control substrates. Specifically, we used vertically oriented Si NWs on a Si wafer with a 10 nm-silica coating (ROC of ∼60 nm), designed to support lightguiding at wavelengths matching the fluorescence emission of Cyanine 5 (Cy5) around 670 nm^39^ (see Section 1, Supporting Information) and conventional glass slides as the planar silica control.

To facilitate the fluorescent visualization of vesicle adsorption and SLB formation, a small fraction (∼1%) of POPC (1-palmitoyl-2-oleoyl-glycero-3-phosphocholine) lipid vesicles, with a mean diameter of 118 ± 4 nm and a polydispersity index of 0.33, were labeled with 1% fluorescent Cy5-DOPE (1,2-dioleoyl-*sn*-glycero-3-phosphoethanolamine-N-Cy5) lipids (see Section 2, Supporting Information), hereafter referred to as fluorescent vesicles. To control the rate of lipid vesicle deposition, planar controls and NW platforms were mounted separately in custom-made soft-lithography microfluidic channels (see Section 3, Supporting Information), enabling pressure-driven liquid flow and rapid (<1 s) liquid exchange. The design of the channel enabled time-resolved lipid vesicle adsorption measurements on the planar control using TIRF and the NW platform using epifluorescence microscopy (Figure 1).

**Figure 1:**
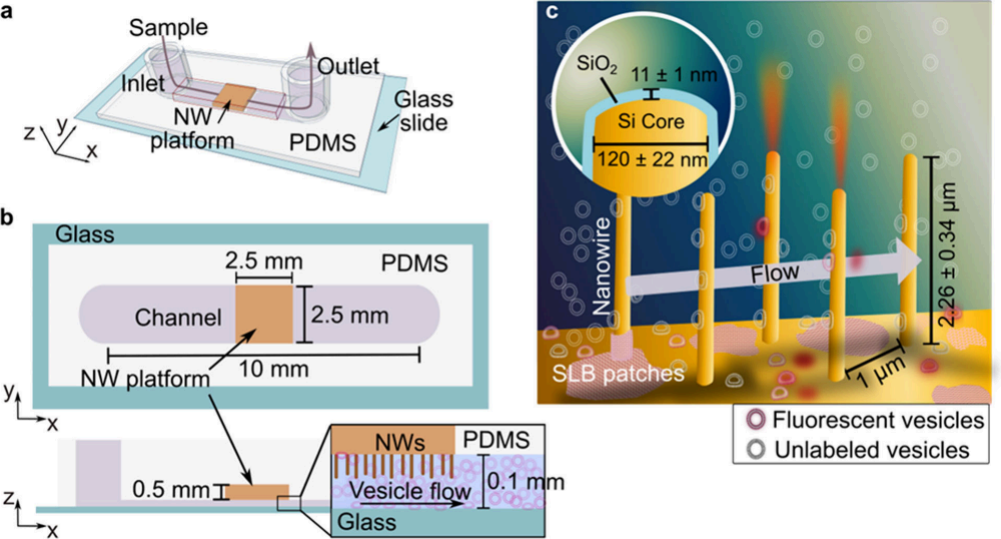
(a) Custom-made microfluidic channel defined by a molded, cross-linked polydimethylsiloxane (PDMS). The device is sealed irreversibly to a glass slide, with the liquid flowing from the inlet to the outlet, driven by negative pressure. (b) Top and side view of the microchannel (10 × 2.5 × 0.1 mm^3^) containing a cavity matched in size to the dimension of the NW platform (2.5 × 2.5 × 0.5 mm^3^), ensuring that only the NWs protrude from the channel top wall. More details on the device design and fabrication are included in Section 3 of the Supporting Information (Figure S1). Glass slides served as the planar silica control in identically dimensioned channels. (c) Graphic representation of the formation of an SLB on lightguiding NWs (not to scale), illustrating continuous vesicle adsorption, and on both the NWs and the floor in-between, causing surface-induced vesicle deformation.

The micrographs in Figure 2 represent the temporal evolution of SLB formation on an array of hexagonally arranged vertical silica-coated Si NWs (Figure 2a) and a planar silica glass surface (Figure 2b). The formation of continuous and homogeneous SLBs was verified using fluorescence recovery after photobleaching (FRAP) measurements,^38^ revealing a diffusivity of 1.29 μm^2^/s and 1.45 μm^2^/s with defect densities of 11% and 4% on the NWs and the planar surface, respectively (see Section 4, Supporting Information, and Movies S1 and S2).

**Figure 2:**
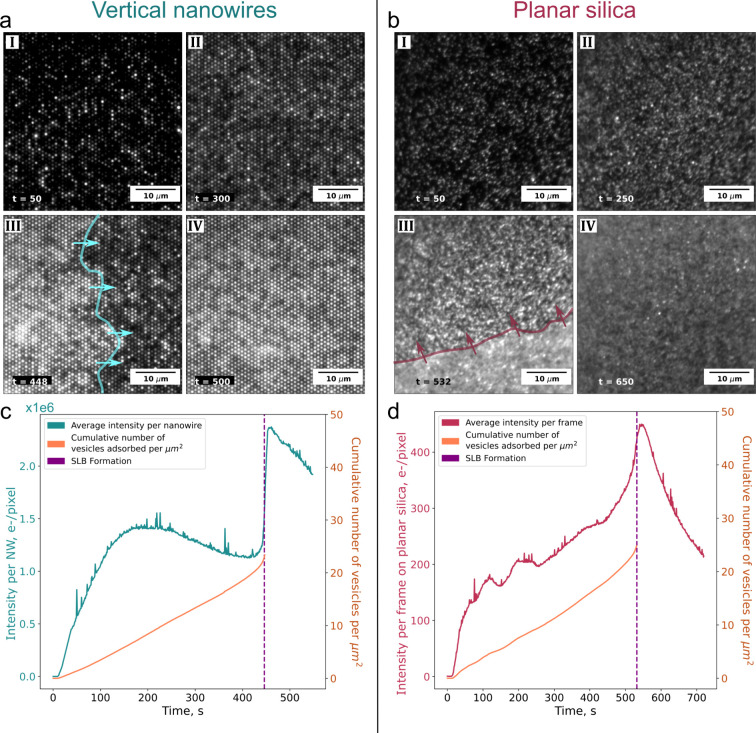
(a) Sequential micrographs (I to IV), depicting critical stages of the SLB formation process on NW platforms imaged by epifluorescence microscopy with the focal plane positioned at the NW tips and (b) on planar silica captured by TIRF microscopy. The process is monitored during continuous addition of a vesicle suspension containing 1% fluorescently labeled lipid vesicles. Micrographs III in a) and b) are snapshots illustrating the onset of SLB formation on the respective surfaces. (c, d) Time evolution of a cumulative number of adsorbed vesicles per μm^2^ (orange) up to the onset of SLB formation, estimated by counting new vesicles emerging upon subtraction of consecutive frames. These graphs also display the time evolution of the background-corrected average intensity per bright NW (turquoise) in (c) and per frame on planar silica (red) in (d) versus time. The data reported in (c) and (d) was obtained with the same image acquisition parameters and microscopy setup, but the fluorescence enhancement of lightguiding NWs results in several orders of magnitude higher signal than on the planar control. The full sequence of micrographs can be found in Section 7, Supporting Information (Movies S3 and S4).

The lightguiding properties and vertical orientation of the NWs result in directional fluorescence emission at the NW tips, indicating the presence of at least one fluorescent vesicle or dye-labeled lipid on their surface.^39^ Upon initial lipid vesicle adsorption, there is a gradual increase in the number of bright NWs (micrographs I and II in Figure 2a), mirrored by a gradual increase in lipid vesicle coverage on the planar control (micrographs I and II in Figure 2b). In both cases, the onset of SLB formation results in an increase in fluorescence intensity and the formation of SLB fronts (micrograph III in Figures 2a and b) that traverse the surfaces.

While SLB formation on the planar control proceeds at a rate (1.4 ± 0.3 μm/s) consistent with previous results,^10,11^ the SLB front on the NW platform advances more than three times faster (4.6 ± 0.8 μm/s). This difference is further reflected in the significantly more rapid increase in total fluorescence intensity at the onset of SLB formation (Figure 2c and d), also verified using glass slides coated with 10 nm silica, as used for the Si NWs. Despite this striking difference, both the rate of newly adsorbed vesicles per unit area and the critical vesicle coverage at which the SLB process is initiated are essentially identical for both substrates (Figure 2c and d). Thus, the striking difference in the speed of the SLB front is not related to differences in the critical vesicle coverage at which SLB formation occurs, which is commonly a signature for how efficient the SLB formation process is for different types of surfaces or lipid compositions.^11,44^

The significant increase in fluorescence emission intensity upon SLB formation (Figure 2c and d) is attributed to evanescent fields generated by lightguiding nanowires and TIR on the planar surface, suggesting that in this step fluorophores move closer to high-intensity regions near the nanowire surface and TIR-illuminated planar silica control. The slightly greater increase observed for NWs compared to the planar control is attributed to the merging of dye-labeled lipids that prior to the onset of SLB formation are bound to the planar floor between NWs, where they before SLB formation experience less photobleaching due to high optical absorption of the NWs.^38^ This interpretation is also consistent with the lower rate of photobleaching observed for the NW surface after completed SLB formation, being attributed to fluorescently labeled lipids within the laterally mobile SLB that are transiently illuminated only when present on the NW.

It is also noteworthy that the average fluorescence intensity exhibits a decrease prior to SLB formation on the NWs (Figure 2c), presumably due to photobleaching, whereas a gradual increase is observed on the planar silica control (Figure 2d). This difference may be attributed to subtle variations in the initial stage of surface-induced vesicle collapse and merging into SLB patches, henceforth inspected by utilizing the capacity of lightguiding NWs and TIRF microscopy to resolve not only individual lipid vesicles containing multiple fluorescently labeled lipids, but also individual dye labeled lipids.

To monitor SLB formation through individual dye-labeled lipids, experiments similar to Figure 2 were conducted, but the adsorption of fluorescent lipid vesicles was in these experiments interrupted at a low coverage (<1 vesicle/μm^2^), significantly below the critical threshold (∼20 vesicles/μm^2^) for SLB formation (Figures 3c and d). To complete SLB formation, unlabeled vesicles were subsequently added, eliminating photobleaching complications and allowing visualization of individual vesicle collapse without interference from new fluorescent vesicles.

**Figure 3:**
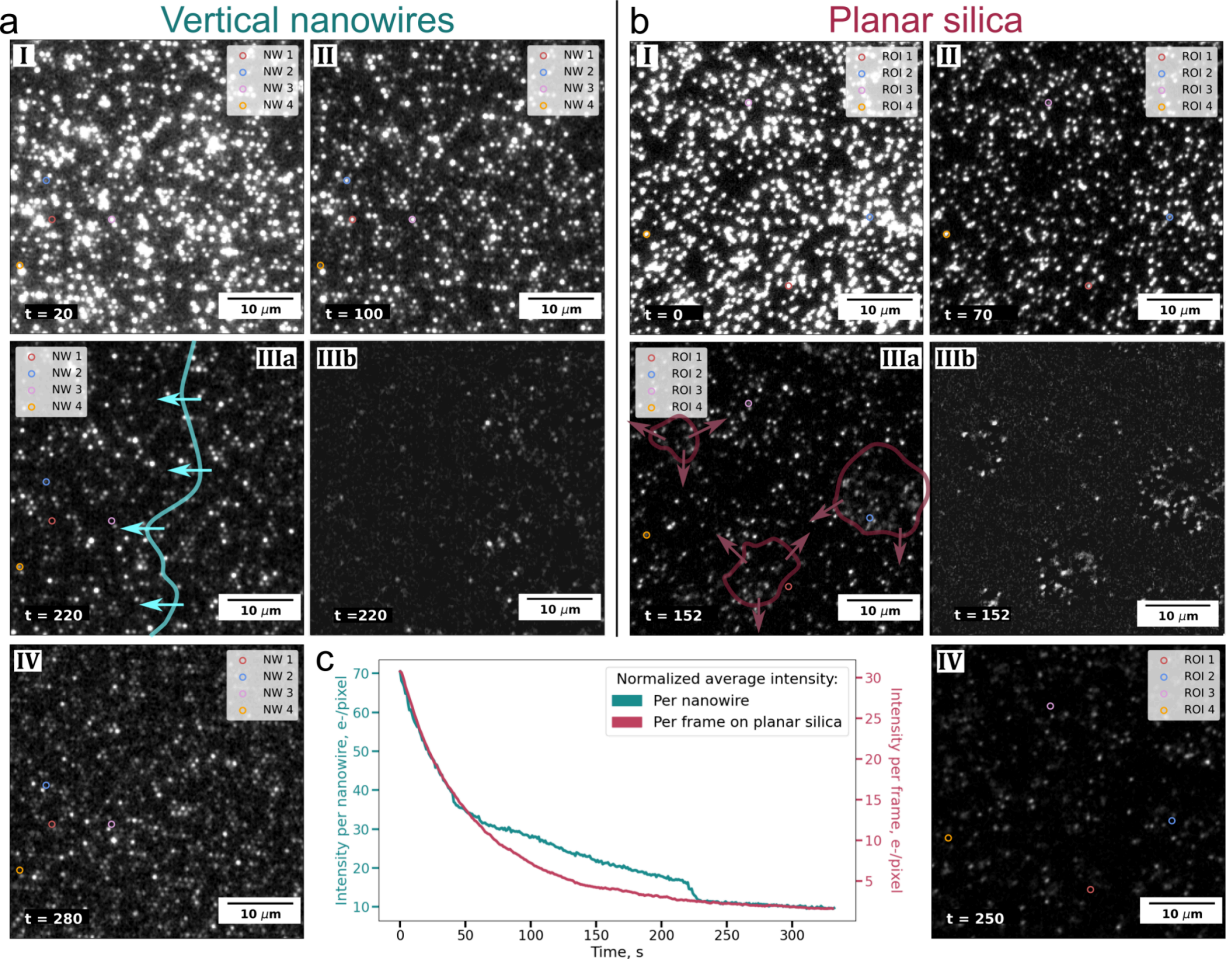
Sequence of micrographs of the SLB formation process on (a) NW arrays and (b) planar control, acquired with epifluorescence and TIRF microscopy, respectively, with initially adsorbed fluorescent vesicles followed by continuous delivery of nonfluorescent vesicles at *t* = 0. Micrographs IIIb in a) and b) correspond to the same frames as in IIIa in a) and b), respectively, depicting the onset of the SLB formation with enhanced contrast, generated by subtracting the intensity of two consecutive frames. Note that the low concentration of laterally mobile, dye-labeled lipids prevents all NWs from simultaneously emitting fluorescence upon completion of the SLB as seen in micrograph IV in a), unless enough images captured after the SLB formation are averaged (Figure S3). The patches appear as regions of fluctuating fluorescence emission (bright and dark spots). (c) Normalized background-subtracted average intensity versus time for bright NWs (turquoise) and the planar control (red) for the entire stack of acquired frames (1 s per frame). The data reported in (c) was obtained using the same image acquisition parameters and microscopy setup. The full sequence of micrographs can be found in Section 7, Supporting Information (Movies S7 and S8).

Micrographs representing this experimental approach are depicted in Figures 3a and b for NWs and the planar control, respectively. As seen in micrographs I, II, and IIIa in Figure 3a, both the initial number of bright NWs and their intensity decrease due to photobleaching of adsorbed vesicles, and similarly, the number of vesicles that remains bright on the planar control gradually diminishes (micrographs I, II and IIIa in Figure 3b). Despite the continuous bleaching process, SLB formation was verified in separate FRAP measurements, revealing diffusion constants of 1.17 and 1.44 μm^2^/s and defect densities of 10 and 12%, for the NW platform and the planar control, respectively (see Section 4.1, Supporting Information, and Movies S5 and S6).

To visualize the onset of SLB formation, enhancing the image contrast by subtracting the preceding frame from each new frame was essential, enabling inspection of the advancement of an SLB front from right to left (Figure 3a., IIIb) for the NWs, as well as the local formation of SLB patches on the planar silica surface (Figure 3b, IIIb), which subsequently merge into an advancing SLB front as shown in Figure 2.

The time evolution of average intensity for the planar silica surface shows a single exponential decay during vesicle adsorption and SLB formation, while the NW platform exhibits a similar photobleaching rate for the first 50 s, in both cases attributed to bleaching of adsorbed vesicles. However, after the initial phase for the NW platform, the photobleaching rate drops by more than a factor of 2, until the onset of SLB formation at around *t* = 220 s (Figure 3c). One plausible interpretation for the reduced photobleaching rate is the early formation of small-scale SLB patches on both the NWs and the planar floor in-between the NWs, where dye-labeled lipids moving within these patches experience reduced photobleaching as they are predominantly excited when present on the NWs. An apparent reduction in the rate of photobleaching may also originate from fluorescent lipids in SLB patches formed from vesicles adsorbed on the floor between the NWs, which were nondetectable prior to SLB patch formation.

It is also worth noting that the actual SLB formation on the NWs is characterized by a rapid drop in intensity within a time scale of 10 s. While this time scale compares to the SLB formation on NWs in Figure 2a, the reduction in intensity, contrary to the increase observed in Figure 2c, is attributed to the spreading of laterally mobile dye-labeled lipids across the entire NW platform, with the NW turning bright only when a laterally mobile dye labeled lipid is present at its interface (see Figure S3).

To scrutinize whether small-scale SLB patches coexisting on both the NWs and the planar floor in-between the NWs appear prior to the onset of global SLB formation process, the time evolution of the fluorescence emission from individual NWs was compared with the temporal evolution of the intensity from individual bright vesicles on the planar silica control, as illustrated with four representative examples in Figures 4a and b, respectively.

**Figure 4:**
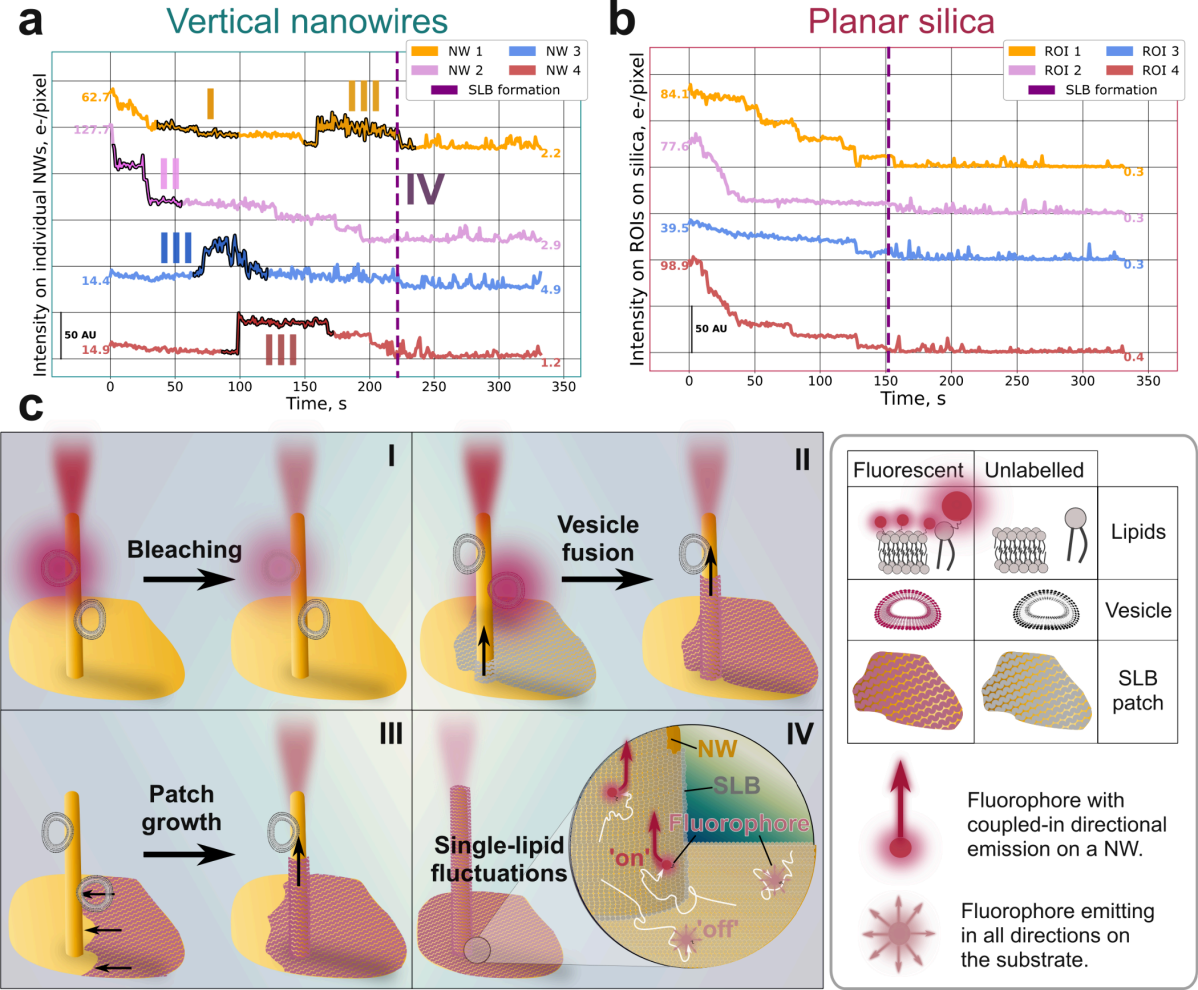
Background-subtracted intensity emission versus time for (a) the individual NWs highlighted in panel Figure 3a, each corresponding to a total surface area of ∼0.9 μm^2^, and (b) the color-coded ROIs marked in panel Figure 3b, each representing an area of 5 × 5 pixels (0.81 μm^2^) similar to that of a NW. In (a) and (b), trace intensity changes are relative, and the labels indicate the initial and final values for comparison. (c) Schematic representation of the intensity evolution featured in (a): (I) gradual decrease in emission intensity, attributed to bleaching of fluorescent lipid vesicles on a NW, see NW 1; (II) instantaneous and drastic drop in fluorescent emission, attributed to vesicle collapse, and merge with an SLB patch devoid of fluorescent lipids, primarily localized on the floor between the NWs, see NW 2; (III) initially dark NW that suddenly becomes bright, attributed to the growth of a fluorescent dye containing SLB patches merging with an unlabeled vesicle on the NWs prior to the onset of complete SLB formation at *t* = 220 s, see NWs 1, 3 and 4; (IV) rapid signal fluctuations on essentially all NWs upon complete SLB formation at *t* = 220 s, attributed to laterally mobile fluorescent lipids diffusing on and off the NWs within an SLB that covers both the NWs and the floor.

NWs 1 and 2 display intensity values well above the background level at the start of the imaging, indicating the presence of at least one dye-labeled vesicle absorbed on these NWs. For NW 1, the intensity initially decreases gradually, attributed mainly to photobleaching (schematically illustrated in graph (I) in Figure 4c). A similar trend is observed for NW 2, for which the intensity decreases stepwise until the onset of SLB formation at *t* ∼ 225 s, attributed to a combination of photobleaching, vesicle collapse and local SLB patch formation dominated by unlabeled lipids, merging with bleached and unlabeled lipids from the initially adsorbed vesicles on the NW (schematically illustrated in graph (II), in Figure 4c). A contrasting behavior is observed for NWs 3 and 4 (highlighted in red and blue in Figure 4a), which initially exhibit intensity values near the background level, suggesting the absence of fluorescent lipid vesicles. However, even though only unlabeled lipid vesicles are introduced to drive the SLB formation process, these NWs experience a drastic intensity increase at *t* = 50 s (NW 3) and *t* = 100 s (NW 4), that is, prior to the onset of global SLB formation at *t* ∼ 225 s. A similar increase is observed for NW 1 at *t* ∼ 150 s, being consistent with the collapse of nearby fluorescent lipid vesicles, likely located on the floor near the base of the NWs, and subsequent formation of SLB patches reaching the examined NWs (schematically illustrated in graph (III) in Figure 4c). Further, the sudden intensity increases for NWs 1, 3, and 4 prior to the SLB formation, accompanied by intensity fluctuations, likely indicate local patch formation with a low number of dye-labeled lipids diffusing in and out of the evanescent field of the NWs (Figure 4c, graph IV). Eventually, all NWs experience a decrease in intensity upon SLB formation, with rapid fluctuations observed for all individual NWs.

In stark contrast, the intensity emission from individual vesicles on the planar control does not display rapid fluctuations until the SLB formation is completed, but rather monotonic step-like intensity reductions, attributed to a single-fluorophore bleaching (Figure 4b). For a comparison with fluorescence time evolution of individual NWs and local region of interests (ROIs) on the planar control for the SLBs shown in Figures 2a and b (see Figure S4). Together, these observations suggest that SLB formation on the two substrates differ not only with respect to the progression of the SLB spreading across the surface but also confirm that the nanowires trigger local small scale patch formation prior to the onset of global SLB formation.

Our results for the planar control confirm the emerging view of the SLB formation process, being initiated by spontaneously adsorbed vesicles that initially undergo deformation upon adsorption, the magnitude of which being determined by the strength of the vesicle-surface attractive adhesion and the energetic penalty associated with membrane bending.^44^ As the surface coverage of vesicles gradually increases, a critical point is reached at which lateral interaction between multiple vesicles induces a collapse into small-scale SLB patches, previously verified by probing single vesicles containing dye-labeled lipids^11,14^ as well as encapsulated dyes.^10^ This surface coverage at which SLB patch formation occurs remains independent of vesicle concentration in the bulk solution,^45^ and due to the stochastic nature of the vesicle adsorption process, microscopic SLB patches emerge randomly on the surface (see micrograph IIIb, Figure 3b), which gradually merge and spread across the surface as the energetically unfavorable edges of the SLB patches interact with already adsorbed and newly adsorbed vesicles.^14,15,46^

In contrast, the analysis of the overall bleaching rate for the NW platform (Figure 3c) and the time evolution of the fluorescence emission from individual NWs (Figure 4a) suggest that small-scale SLB patches emerge significantly earlier than the onset of global SLB formation. The observed small-scale SLB-patch formation, verified from sudden changes and fluctuations in local intensity (Figure 4a), is likely to be facilitated by the rupture of vesicles adsorbed at the concave region at the foot of the NWs, offering an increased contact area, and thus more pronounced surface-induced vesicle deformation.^43^ Further, the membrane bending penalty of an SLB formed on a cylindrical NW is expected to be lower than that of a deformed vesicle, which is also likely to favor vesicle collapse. Considering the stark difference in the rate of progression of the SLB front across the two types of substrates (Figure 2), it is worth noting that the growth of a SLB patch on a planar substrate must overcome the penalty associated with expanding the energetically unfavorable rim of the patch,^10,11^ while the length of a rim of an SLB patch that progresses along the axis of a NW will remain essentially constant. Thus, the progression of the SLB along the nanowire surface is anticipated to be energetically favorable. Although it is not immediately obvious how this aligns with the observed high rate of lateral expansion across the NW substrate, the expansion of the SLB patch along the NW involves lipid material from vesicles adsorbed on the NW surface merging with the SLB. The available lipid material on a NW can be estimated from the vesicle coverage of around 30% at the onset of global SLB formation (Figure 2c), which means that vesicle collapse into a planar shape corresponds to a 20% surplus of lipid material. Thus, as soon as the SLB patch covers the entire NW, this additional lipid material must escape at the foot of the NW, contributing to the observed faster advancement of the SLB front on the NW substrate compared with the planar surface. Hence, due to a combination of small-scale SLB patch formation promoted by the NW geometry and favorable SLB growth along the NW axis, our results suggest that vertically aligned high aspect ratio NWs facilitate SLB formation, contrary to previous findings that indicated hampered SLB formation on nanostructured interfaces.^42^

Owing to the NW fluorescence-enhancing properties, our results also suggest that lightguiding NWs may serve as ideal probes for investigating the mobility of lipids and other membrane-associated molecules. To exemplify this potential of the SLB-coated NWs, fluorescently labeled streptavidin (A647-Stv) was specifically bound to biotin-modified lipids in an SLB formed on the NW substrate, followed by time-resolved imaging of individual nanowires (Figures S5 and S6, Section 6, in Supporting Information). From inspection of the temporally resolved intensity variation of individual nanowires, it is evident that one can extract information not easily obtained by other means, including (i) the average time between two bursts on the same nanowire, which corresponds to the time scale of A647-Stv diffusion on the planar floor between the nanowires, and (ii) the duration of the bursts, which represents the residence time of A647-Stv on the highly curved nanowires. Although beyond the scope of this work, this possibility to disentangle protein diffusivity on planar and highly curved surfaces is particularly relevant when analyzing curvature-sensitive proteins and could help unraveling mechanistic details regarding how membrane curvature sensing and remodeling control critical cellular processes and even disease progression.^21,47^

## Supporting Information Available

(PDF) SLB formation for Si NWs (Movies S3 and S7) and planar silica (Movies S4 and S8). (AVI) FRAP tests for Si NWs (Movies S1 and S5) and planar silica (Movies S2 and S6). (AVI) The Supporting Information is available free of charge at https://pubs.acs.org/doi/10.1021/acs.nanolett.4c05303.

Further information on the growth of Si NW platforms and SEM morphological characterization, description of the surface treatments for both Si NWs and planar silica surfaces, the preparation of vesicle suspensions and their characterization, the fluorescence microscopy setup used, a protocol for the preparation of the custom-made microfluidics device and its detailed blueprints, a description of the experimental setup and the calculations for performing FRAP test on the formed SLBs, the intensity-averaged stack of frames after the SLB formation to show the lateral mobility of lipids, complementary measurements of the intensity of individual NWs and ROIs in planar silica from the experiments shown in Figure 2, and complementary results of the temporally resolved intensity variation of individual NWs for proteins bound to the SLB (PDF)
FRAP of the SLB shown in Figure S2a (AVI)
FRAP of the SLB shown in Figure S2b (AVI)
SLB formation on Si NWs (data shown in Figure 2a) (AVI)
SLB formation on planar silica (data shown in Figure 2b) (AVI)
FRAP of the SLB in Figure S3a (AVI)
FRAP of the SLB in Figure S3b (AVI)
SLB formation on Si NWs (data shown in Figure 3a) (AVI)
SLB formation on planar silica (data shown in Figure 5) (AVI)

## Supplementary Material

nl4c05303_si_001.pdf

nl4c05303_si_002.avi

nl4c05303_si_003.avi

nl4c05303_si_004.avi

nl4c05303_si_005.avi

nl4c05303_si_006.avi

nl4c05303_si_007.avi

nl4c05303_si_008.avi

nl4c05303_si_009.avi

## ABBREVIATIONS

SLB: supported lipid bilayer
ROC: radius of curvature
Si: silicon
NW: nanowire
TIR(F)(M): total internal reflection (fluorescence) (microscopy)
AFM: atomic force microscopy
iSCAT: interferometric scattering
POPC: 1-palmitoyl-2-oleoyl-glycero-3-phosphocholine
DOPE-Cy5: 1,2-dioleoyl-*sn*-glycero-3-phosphoethanolamine-N-(Cyanine 5)
PDMS: polydimethylsiloxane
FRAP: fluorescence recovery after photobleaching
ROI: region of interest
LNP: lipid nanoparticle
