# Supplementary material for: Comparative Kinetics of Supported Lipid Bilayer Formation on Silica Coated Vertically Oriented Highly Curved Nanowires and Planar Silica Surfaces
Source: Nano Lett. 2025 Feb 6;25(8):3085–92. doi: 10.1021/acs.nanolett.4c05303 (PMC11869362; doi:10.1021/acs.nanolett.4c05303)
Supplement: Supplementary file 1 — nl4c05303_si_001.pdf [file nl4c05303_si_001.pdf]

Supporting Information

Comparative Kinetics of Supported Lipid Bilayer Formation on Silica Coated Vertically Oriented Highly Curved Nanowires and Planar Silica Surfaces

*Julia Valderas-Gutiérrez,[1,2] Rubina Davtyan, [1,2] Christelle N. Prinz, [1,2] Emma Sparr, [3] Peter Jönsson, [3] Heiner Linke, [1,2] and Fredrik Höök.[1,4]**

[1]NanoLund, Lund University, P.O. Box 118, SE-22100 Lund, Sweden

[2]Solid State Physics, Lund University, P.O. Box 118, SE-22100 Lund, Sweden

[3]Physical Chemistry, Lund University, P.O. Box 124, SE-22100 Lund, Sweden.

[4]Department of Physics, Chalmers University of Technology, SE-41296 Göteborg, Sweden.

TABLE OF CONTENTS:

Section 1. Growth and characterization of vertical arrays of Si NWs **3**

1.1. Plasma etching growth of Si NWs *3*

1.2. Properties of Si NW platforms *4*

Section 2. Formation of supported lipid bilayers **5**

2.1. Vesicle preparation via sonication *5*

2.2. Surface treatment of planar silica and Si NWs *5*

2.3. SLB formation on planar silica and Si NWs *6*

2.4. Optical fluorescence microscopy *6*

Section 3. Custom-made microfluidic device **8**

Section 4. Studies of the lateral mobility of lipids on the formed SLB **10**

4.1. Fluorescence recovery after photobleaching *10*

4.2. Lateral motion of lipids captured in an intensity-averaged image *12*

Section 5. Time evolution of the intensity of individual NWs and ROIs in Fig. 2 **14**

Section 6. Temporally resolved intensity variation of individual NWs for streptavidin bound to the SLB **16**

Section 7. Complementary movies **19**

Section 1. Growth and characterization of vertical arrays of Si NWs.

This section details the growth of the Si nanowires (NWs) used in this work and their properties.

1.1. Plasma etching growth of Si NWs.

Silicon (Si(100)) wafers were patterned by displacement Talbot lithography (DTL), starting with the deposition of a double layer of developable resists: a bottom antireflection coating or BARC (SF 3S) and a deep-ultraviolet resist or DUVR (PAR1085S90). An additional shrink resist (AZ-SH-114A) was used to reduce the diameter of the pattern holes. The pattern was transferred to the resists using PhableR 100 deep ultraviolet (DUV) equipment (EULITHA AG, Switzerland). This defined a hexagonal pattern of 120 nm holes with 1 µm pitch with a density of 1.19 μm^2.

Chromium seed nanoparticles (~105 nm in diameter) were deposited through e-beam evaporation followed by resist removal (lift off) in an acetone bath at 100 ºC. Si NWs were grown from the seed nanoparticles by anisotropic dry plasma etching. Details of the etching process belong to proprietary information owned by the Technical University of Denmark (DTU). These are not disclosed but are comparable to standard Si etching procedures as described elsewhere.[1] Additionally, the etched Si NWs were coated with 10 nm of SiO_2 using atomic layer deposition (ALD, Fiji from Veeco, USA). As detailed below, a morphological characterization was performed by scanning electron microscopy (SEM, Leo System, Zeiss, Germany).

After growth, the full wafer was coated with a protective photoresist to avoid damage while diced into identical squared pieces of 2.5×2.5 mm^2 using a DAD 3320 Dicer, from DISCO, Germany. To remove the photoresist and other organic and inorganic contaminants, a thorough wash and a plasma treatment were applied, described in detail in Section 2.2.

1.2. Properties of Si NW platforms.

SEM was used to measure the dimensions of the Si NWs, defined by the widest (d_W) and thinnest diameters (d_T) in the absence of SiO_2 coating, the length (L), and the thickness (t_c) of the SiO_2 coating, for a sample of $N \geq 30$ NWs. The average diameter (d_A) was estimated as $(d_W + d_T)/2$. The tapering factor is calculated as $(d_W - d_T)/(d_W + d_T)$.

The NW density or coverage (c_{NW}) was defined by the hexagonal DTL pattern of Si NWs and evaluated by SEM. Only the density of non-defective or non-kinked NWs was considered (99 %), yielding a final value of 1.17 μm^{-2}. The lateral surface area per NW (A_{NW}) was calculated using a formula applicable for a truncated cone: $A_{NW} = \frac{\pi}{2}(4t_c + d_W + d_T)\sqrt{\frac{1}{4}(d_W - d_T)^2 + L^2}$. The effective surface area (A_E) corresponded to the NW area per substrate area, obtained from $A_{NW} \times c_{NW}$, defining the additional NW surface area that exists for molecular binding in 1 μm^2 of the substrate, which does not exist in a planar substrate without nanostructures:

d_W (nm)	120 ± 22
d_T (nm)	100± 11
d_A (nm)	110 ± 12
L (µm)	2.26 ± 0.34
t_c of SiO_2 (nm)	11 ± 1
Spacing (µm)	0.99
c_{NW} (NWs/ μm^2)	1.17
Tapering factor	0.032
Tilt away from vertical (degrees)	Negligible
A_{NW} (μm^2/NW)	0.93 ± 0.13
A_E (μm^2/ μm^2)	1.08 ± 0.16

Table 1. Summary of the most relevant morphological properties of the Si NWs used in this work, characterized using SEM, for a sample of $N \geq 30$.

Section 2. Formation of supported lipid bilayers.

2.1. Vesicle preparation via sonication.

A lipid stock solution of 1-palmitoyl-2-oleoyl-glycero-3-phosphocholine, abbreviated as POPC (16:0 – 18:1 PC) from Avanti Polar Lipids, USA, was used for the preparation POPC vesicles. Dye-labeled vesicles were made by mixing 1,2-dioleoyl-sn-glycero-3-phosphoethanolamine-N-(Cyanine 5) abbreviated as DOPE-Cy5 (18:1 PE), and POPC in chloroform (VWR, USA) at a molar ratio of 1:99. The solvent was evaporated under a nitrogen stream and the dried lipids were resuspended in HEPES Buffer Saline, HBS (150 mM of NaCl and 10 mM of HEPES (both from Merck, Germany) in Milli-Q water at pH 7.2), to a final lipid concentration of 0.5 mg/ml. The heterogeneous vesicle suspension was sonicated using a tip sonicator (VCX 130, Sonics & Materials, USA) for 30 minutes at 65 W in on-off pulses of 10 seconds until a final homogeneous and visibly transparent suspension was obtained. When prepared, the suspension of dye-labeled vesicles was further mixed with pure POPC vesicles at 1:100 molar ratio (hereafter referred to as tracer vesicles suspension), to ensure that only 1% of the vesicles were fluorescently labeled.

The refractive index and the viscosity of the buffer were calculated with the research tool SEDNTERP170,[2] and shown to be indistinguishable from water. Dynamic light scattering (DLS) measurements revealed that POPC vesicles had a diameter of 118 ± 4 nm with a polydispersity of 0.33, measured with a Malvern Zetasizer Nano ZS (Malvern Instruments, UK), using a 640 nm laser.

2.2. Surface treatment of planar silica and Si NWs.

Both silica and NW substrates require pre-cleaning and activation of the surfaces to facilitate SLB formation. For silica surfaces, glass slides (25×60×0.17 mm^3, 1.5# from Menzel-Gläser, ThermoFisher Scientific, USA) were cleaned in a bath of Piranha solution (mixture of 9 ml of

sulfuric acid and 3 ml of 30 % hydrogen peroxide, both from Merck, Germany) for 30 minutes at 30 ºC. They were later thoroughly washed and stored in Milli-Q water to preserve the activation of the surface, only to be rinsed and dried with a nitrogen stream right before the experiment.

After the dicing of silica coated Si NW platforms, the photoresist, and other remaining organic or inorganic contaminants are removed: Every NW platform was washed sequentially for 30 minutes with first acetone and then isopropanol (both from Merck, Germany) and gently dried under a nitrogen stream. They were then treated under UV-ozone plasma (UV-Ozone Cleaning System UVOH 150, FHR Anlagenbau GmbH, Germany) for 90 minutes and 90 ºC, at 500 mbar. Finally, they were stored in a sealed box, in a clean and dry environment at room temperature until their use.

2.3. SLB formation on planar silica and Si NWs.

For the data shown in Figs. 2a and b, a suspension containing 1% tracer vesicles was diluted 10 times in HBS buffer. A volume of 100 µl was continuously introduced into the microfluidic device at a flow rate of 8 µl/min, while vesicle adsorption was continuously monitored using epifluorescence and TIRF microscopy for the NW and planar substrates, respectively. For Figs. 3a and b, a suspension containing 1% tracer vesicles was diluted 200 times in HBS buffer and 40 µl of the diluted suspension was pumped into the device at a flow rate of 2 µl/min for a total of 2 min. The excess of tracer vesicles was removed from the reservoir and washed thoroughly with 200 µl of HBS (five times). Image acquisition was initiated after adding 100 µl of pure POPC vesicles diluted 10 times in HBS buffer into the device at 8 µl/min until full SLB formation is recorded.

2.4. Optical fluorescence microscopy.

For all the experiments in this work, the imaging parameters used are those described below, unless otherwise stated.

An optical fluorescence microscope (inverted Eclipse Ti2 from Nikon, Japan) equipped with a sCMOS camera (Sona-4BV11 from Andor, Oxford Instruments, UK), and a Cy5 filter cube (Semrock FF01-698/70-Cy5, also from Nikon) was used to monitor the formation of SLBs on all substrates. For planar silica, images were acquired in TIR mode at an angle of 62º, with 60× magnification and an oil immersion objective (Apo TIRF 60x Oil DIC N2, 1.49 NA, Nikon). For the NW samples, epifluorescence mode was used, with 60× magnification and a water immersion objective (Plan Apo VC 60xA WI DIC N2, 1.2 NA, Nikon). The light source was a 640 nm laser, operating at a power of 6 mW, acquiring one frame per second at an exposure time of 100 ms, until the SLB was fully formed.

Section 3. Custom-made microfluidic device.

The design of the microfluidics device designed to be compatible with both epifluorescence and TIRF microscopy of the NW platforms is based on a single microchannel (2.5×10×0.1 mm^3), with a cavity in the middle of the channel with the dimensions of an individual diced NW platform (2.5×2.5×0.5 mm^3), serving as a "pocket" to perfectly fit the substrate, and only expose the NWs to the flow (Fig. S1a). A mold with this design was created with a micro-milling tool (CNC micro-milling, Daltron neo series 2, from Daltron Dynamics, USA), made of polyoxymethylene (POM).

These custom-made devices were prepared by soft lithography using uncured poly(dimethylsiloxane) (PDMS), mixed at a 10:1 ratio with a curing agent (both from the Sylgard™ 184 Silicon Elastomer Kit from Midland, USA), poured on the custom mold and left in the oven for at least 60 minutes to cross-link at 80 ºC (Fig. S1b, graphs I and II).

When solidified, the polymer was peeled off from the mold and cleaned by subsequent washes of isopropanol and water and dried with nitrogen. This was followed by the opening of a 2 mm inlet and outlet at both sides of the channel. The PDMS microchannel was subsequently irreversibly sealed by bonding with the cleaned glass coverslip by activating both surfaces for 10 and 50 seconds, respectively, with air plasma (Plasmatic Systems, USA) (Fig. S1b, graph III).

For the SLB formation on Si NW platforms, they were introduced and glued into the cavity on the channel (Elastosil AO7, RTV-1 silicon rubber, Wacker Chemie, Germany) before sealing, while for planar silica surfaces, the glass used to seal the device served as the sample itself (Fig. S1b, IV). After mounting, the channel was immediately wetted with an aqueous buffer and reservoirs (maximum volume of 200 μl) were glued to the inlets and outlets, made of plastic tubing (3 mm of diameter from Avantor, VWR, USA). Larger tubing (1 mm in diameter Avantor) connected the device to a two-syringe infusion/withdraw pump (SP210IWZ, from World Precision

Instruments, USA) to provide controlled negative pressure-driven flow on the finished device (Fig. S1b, V).

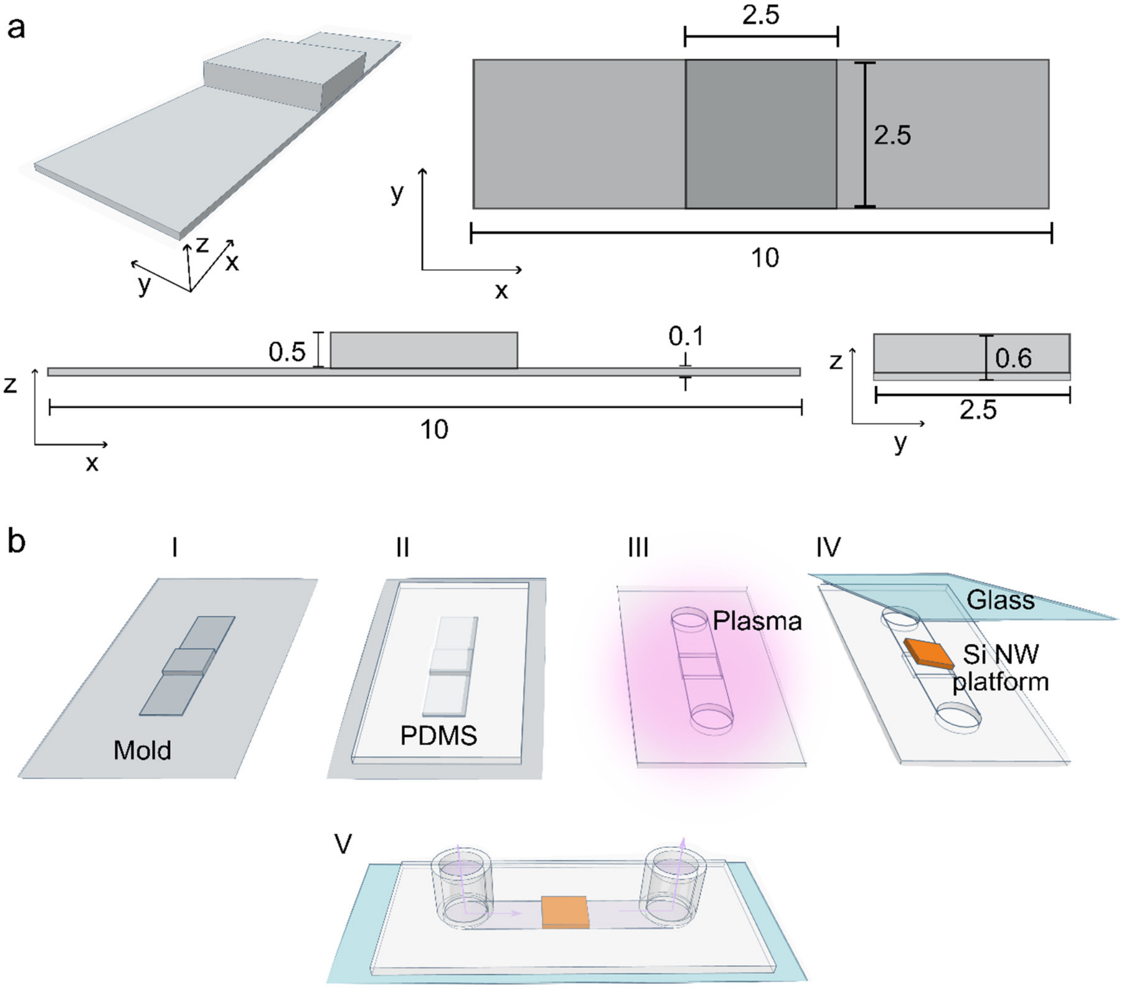

Figure S1. (a) Blueprints of the mold used to create a microchannel of dimensions 2.5×10×0.1 mm^3. A cavity for the Si NW platform of dimensions 2.5×2.5×0.5 mm^3 is included. All the dimensions shown are in millimeters. **(b)** The preparation of the device started with a mold (I) to which the PDMS mixture was poured and cured (II), followed by the activation of the PDMS and silica surfaces with plasma (III), and the attachment of the NW sample on the dedicated cavity (IV) before sealing the channel. On the finished device (V), reservoirs were added, and it was connected to a pump for the controlled delivery of the vesicle solution to the substrate. The device for SLB formation in planar silica surfaces was identical, without the incorporation of a Si NW platform.

Section 4. Studies of the lateral mobility of lipids on the formed SLB

4.1. Fluorescence recovery after photobleaching.

Fluorescence recovery after photobleaching (FRAP) tests were performed before the completion of all experiments to evaluate the homogeneity and lateral mobility of the SLB and to calculate the diffusivity and immobile fraction in each case.

FRAP micrographs were acquired after the SLB was formed. Before the measurements, the sample was rinsed with 200 μl of HBS at a flow rate of 8 μl/min for at least 20 minutes, to wash away the remaining vesicles in the solution.

The fluorescence microscopy setup was identical to the one described in section 2.4. TIRF microscopy was used for planar silica, and epifluorescence microscopy was used for Si NW platforms. Each FRAP experiment had a total duration of 180 seconds, at an exposure time and acquisition rate of 100 msec and one frame per second, respectively. Four initial frames were acquired before bleaching and at t = 5 s, at which a high-intensity laser beam at a power of 100 mW was used to bleach a central circular area of the field of view. The bleached region had a diameter of 25 μm. The fluorescence recovery was measured for the next 180 frames at a laser power of 6 mW.

The changes in fluorescence signal over time in a bleached area of 25 μm in diameter were analyzed to obtain the diffusion constant (D_{Pl}) of the fluorescent molecules in SLBs formed on planar silica. The mean-squared displacement, $\langle r\rangle^2$, depends on D_{Pl} and the recovery time constant, τ_{D}, or the time required for the fluorescence bleached area to recover to 1/2 of the initial intensity, as:[3,4]

$$\langle r\rangle^2 = 4D_{\mathrm{Pl}}\tau_{\mathrm{D}}$$

Here, the relevant displacement r was 12.5 μm, i.e., the radius of the circular bleached area. Several fitting models can be applied to the time evolution of the fluorescence recovery, $F(t)$. For one type of freely diffusing component, the relation between $F(t)$ and τ_D can to a first approximation be expressed as:[5,6]

$$F(t) = \mathrm{A}_1 + \mathrm{A}_2(1 - e^{-t/\tau_\mathrm{D}})$$

where A_1 and A_2 are constants. Using the Levenberg-Marquard[7] algorithm to solve the nonlinear least-squares fitting, it was possible to find A_1, A_2, and τ_D, by utilizing that A_1 can be approximated as the first post-bleach intensity value, and A_2 as the final post-bleach value.[5]

Under the assumption that there is an equal preference of the mobile lipids for both the NW surface and the substrate, the presence of nanostructures on the substrate increases the area available for molecules to diffuse on. For a proper estimate of the diffusion constant for SLBs formed on arrays of NWs, D_{NW}, the average NW diameter d_A, length L, and density, c_{NW}, were considered for a bleached area identical in size to that of the planar substrate. With D_{Pl} obtained as described above, D_{NW} can then be expressed as:[8,9]

$$D_\mathrm{NW} = D_\mathrm{Pl}(1 + \pi d_\mathrm{A} L c_\mathrm{NW})$$

When the intensity values are normalized to the maximum pre-bleaching intensity, the immobile fraction, $I = 1 - \mathrm{A}_2$, is used as a measure of the density of defects on the bilayer.[10,11]

The intensity recovery values and the exponential decay fitting of the data are shown in Fig. S2, from which the following values were obtained: D_{NW} = 1.29 μm²/s and I = 11 % for the SLB presented in Fig. 2a in the main text (Fig. S2a), D_{Pl} = 1.45 μm²/s and I = 4 % for the SLB presented in Fig. 2b in the main text (Fig. S2b), D_{NW} = 1.17 μm²/s and I = 10% for the SLB presented in Fig. 3a in the main text (Fig. S2c), and D_{Pl} = 1.44 μm²/s and I = 12% for the SLB presented in Fig. 3b in the main text (Fig. S2d).

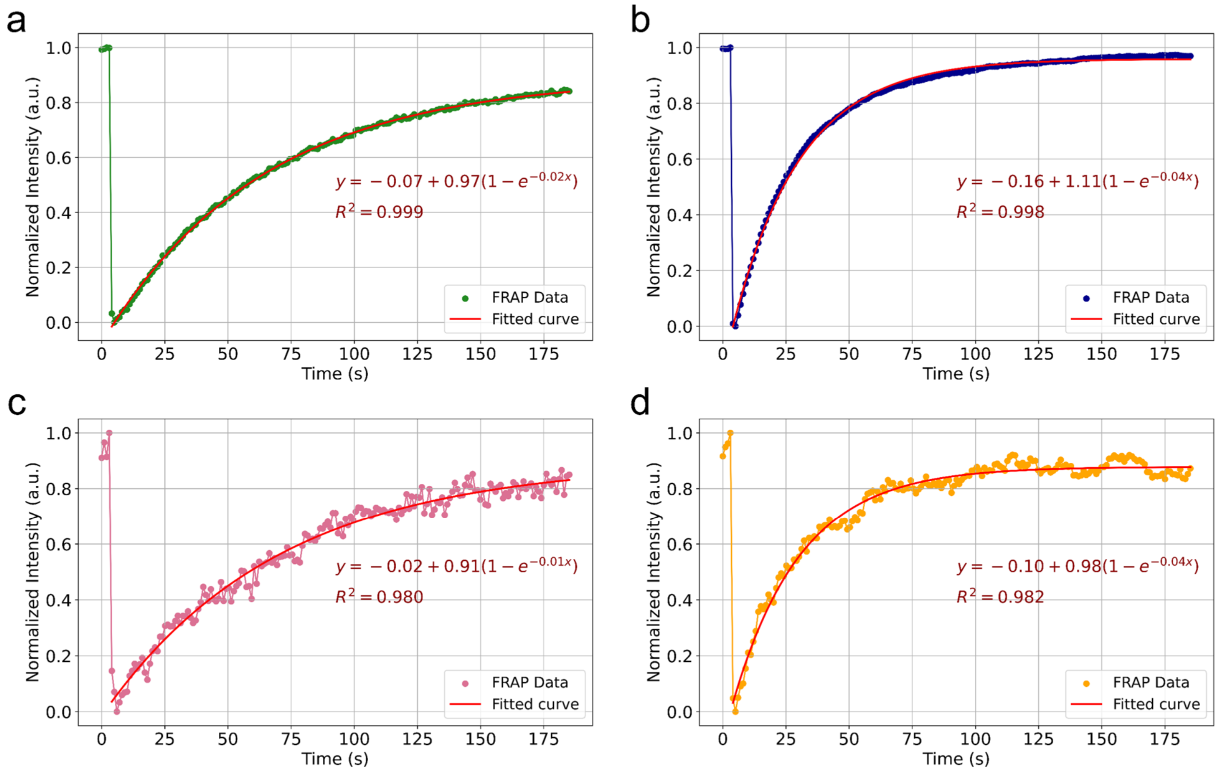

Figure S2. Time evolution of the background-corrected intensity curves after photobleaching of the SLBs analyzed in the main text for **(a)** the data in Fig. 2a, **(b)** Fig. 2b, (**c**) Fig. 3a and **(d)** Fig. 3b. The equation resulted from the exponential decay fitting and the R^2 values are included. The values were normalized to the maximum pre-bleaching intensity. The movies that generated the data in this figure can be found in section 5: Movies 5-8 correspond to panels (a-d), respectively.

4.2. Lateral motion of lipids captured in an intensity-averaged image.

Despite the low concentration of fluorescently labelled dyes in the experiment shown on Fig. 3a, the lateral mobility of lipids on the bilayer can be also observed in an averaged image of no more than 15 frames after the SLB formation on the NWs. As a result, all the NWs on the field of view are perceived as bright (Fig S3a), while a single frame shows only a fraction of bright NWs (Fig S3b).

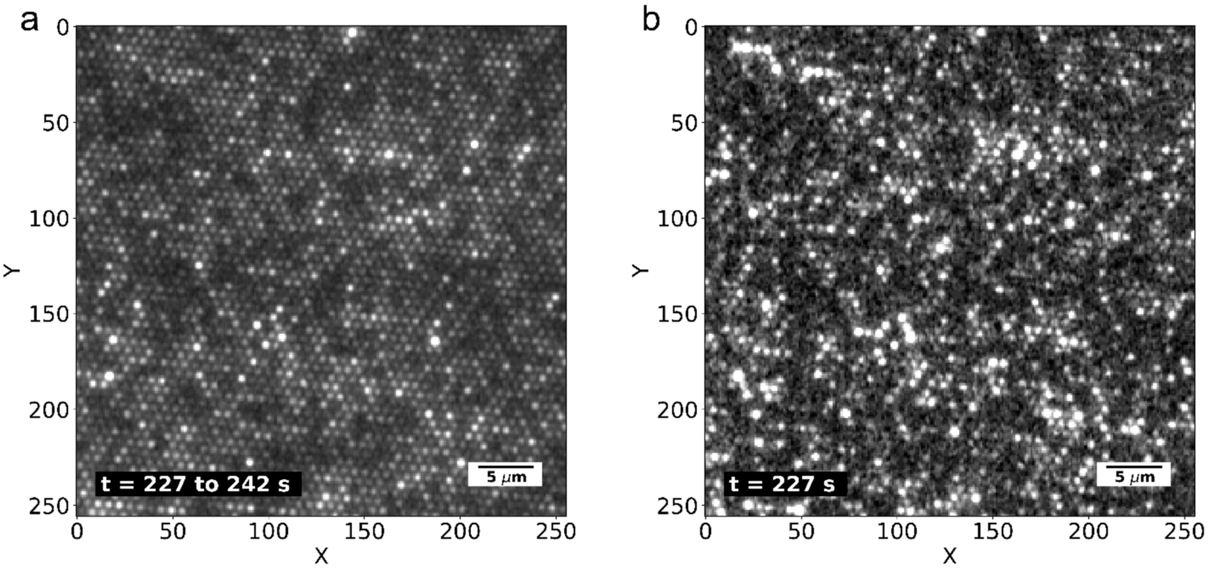

Figure S3. (a) Intensity-averaged image over 15 consecutive micrographs after the formation of the SLB at t = 227 s, which shows the totality of the NWs in the field of view on their hexagonal arrangement. **(b)** Individual micrograph acquired at t = 227 s, with only a fraction of bright NWs and a less homogeneous distribution of the intensity, as a result of the low concentration of fluorescent lipids. The same brightness/contrast parameters are applied in both micrographs for the sake of an accurate comparison.

Section 5. Time evolution of the intensity of individual NWs and ROIs in Fig. 2.

Figure S4c presents fluorescence emission versus time for the data shown in Fig. 2a in the main text, but for four representative NWs (highlighted in Fig. S4a), each with an area of ~0.9 μm². Before the initiation of the SLB formation, these regions exhibit sudden intensity spikes attributed to individual fluorescent vesicle binding events, most of which are followed by gradual photobleaching induced intensity reductions. Upon SLB formation at *t* ~ 448 s, a drastic intensity increase is observed for all NWs, attributed to the rapid collapse of lipid vesicles into an SLB, followed by a gradual intensity reduction due to photobleaching and significantly higher short-term intensity fluctuations than observed prior to SLB formation. These fluctuations are attributed to individual dye-labeled lipids moving within the continuous SLB and on and off the NW, the dynamics of which might also be influenced by local mode patterns of varying excitation intensity along the NW.[12]

Figure S4d presents fluorescence emission versus time for the data shown in Fig. 2b in the main text, but for four ROIs (highlighted on Fig. S4b), each measuring 5×5 pixels / ~0.81 μm². Mirroring the observations on individual NWs, sudden stepwise increases of intensity represent the successive adsorption of several vesicles followed by bleaching. At the onset of the SLB formation, there is an increase in both the signal intensity and the magnitude of temporal fluctuations (*t* ~ 532 s). The short-term fluctuations observed from frame to frame in the images captured after SLB formation are a measure of the movement of SLB-confined dye-labeled lipids as they enter and exit the examined area.

Although the continuous flow of fluorescent vesicles makes it complicated to distinguish between vesicle adsorption events and vesicle rupture followed by patch formation events, the major increase in signal fluctuations could offer an opportunity to quantify both lipid diffusivity and the concentration of dye-labeled lipids as previously described.[13]

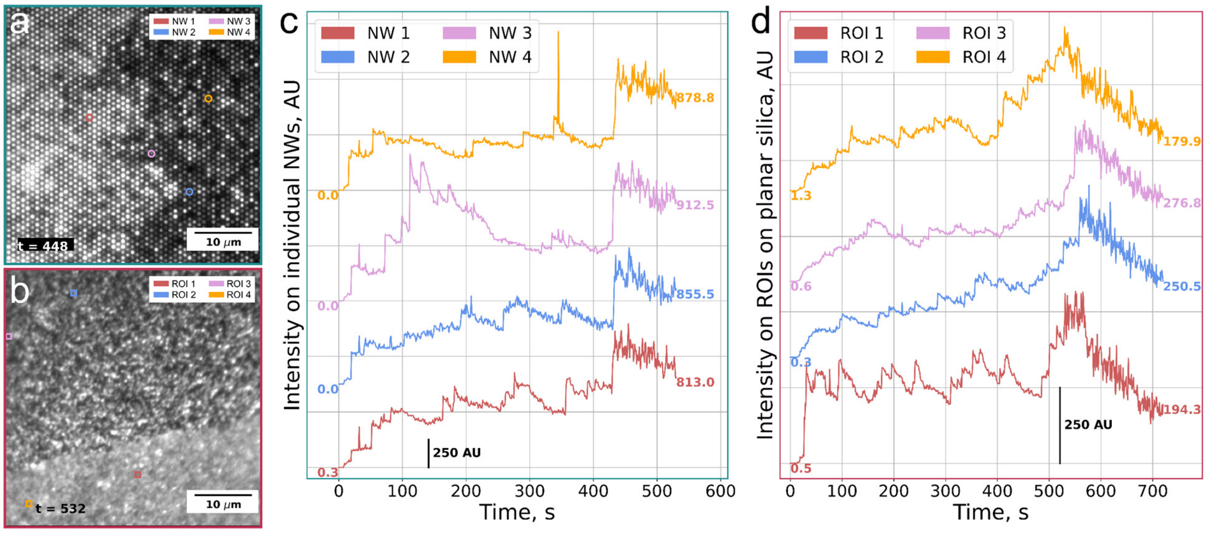

Figure S4. (a, b) Micrographs at the onset of the SLB formation, highlighting four NWs in **(a)** and four ROIs on planar silica in **(b)** from the same experiments as Fig. 2 of the main text. **(c, d)** Intensity evolution over time of the individual NWs **(c)** and ROIs **(d)** previously underlined on **(a)** and **(b)**, showing the sudden intensity changes resulting from the adsorption of vesicles in the examined area, and the high-frequency fluctuations due to the mobility of individual dye-labeled lipids after the SLB formation.

Section 6. Temporally resolved intensity variation of individual NWs for streptavidin bound to the SLB

In this section we provide example data demonstrating the ability to sense and analyze the diffusion of individual proteins bound to the SLB. To compare the intensity fluctuations attributed to individual dye-labeled lipids in the SLB (see Fig. 4a) with the corresponding temporal evolution of individual proteins specifically bound to the SLB, we formed SLBs with an average of 10 biotin-modified lipids per individual NW. This was accomplished by adding a suspension of 100% POPC vesicles mixed with POPC vesicles containing 0.1% biotin-modified lipids (DSPE-PEG(2000)-Biotin) at a 99:1 ratio. The SLB-modified silica-coated Si NW platforms were subsequently incubated with Alexa Fluor 647-labeled streptavidin (A647-Stv) at concentrations of 0.1 and 1 nM. After saturating the biotin-containing SLB with A647-Stv, imaging was conducted for 10 seconds at a rate of 100 frames per second over a 9×9 μm^2 region, using 50% of the excitation intensity employed in the measurements presented in the main text. Snapshots of ten representative temporally resolved intensity fluctuation traces from individual NWs are shown in Fig. S5a and S5b for 0.1 and 1 nM A647-Stv, respectively. These traces are characterized by distinct burst events, attributed to the presence of individual A647-Stv on each NW. From these measurements, one can extract information not easily obtained by other means, including: i) the average number of bursts per NW, scaling with A647-Stv coverage; ii) the average time between two bursts on the same NW, relating to the time scale of A647-Stv diffusion on the planar floor between the NWs; and iii) the duration of the bursts, indicating the residence time of A647-Stv on the NWs. The number of bursts increase with increasing A647-Stv concentration, while the average burst duration (Fig S6a) and mean intensity (Fig. S6b) is independent on concentration, verifying single protein detection at this low protein coverage. This multiparametric information contained in this

type of data presents a significant advantage for various biosensor applications, and may also provide the ability to differentiate protein diffusivity on SLBs when formed on both planar and highly curved geometries, which is particularly relevant when analyzing curvature-sensitive proteins.

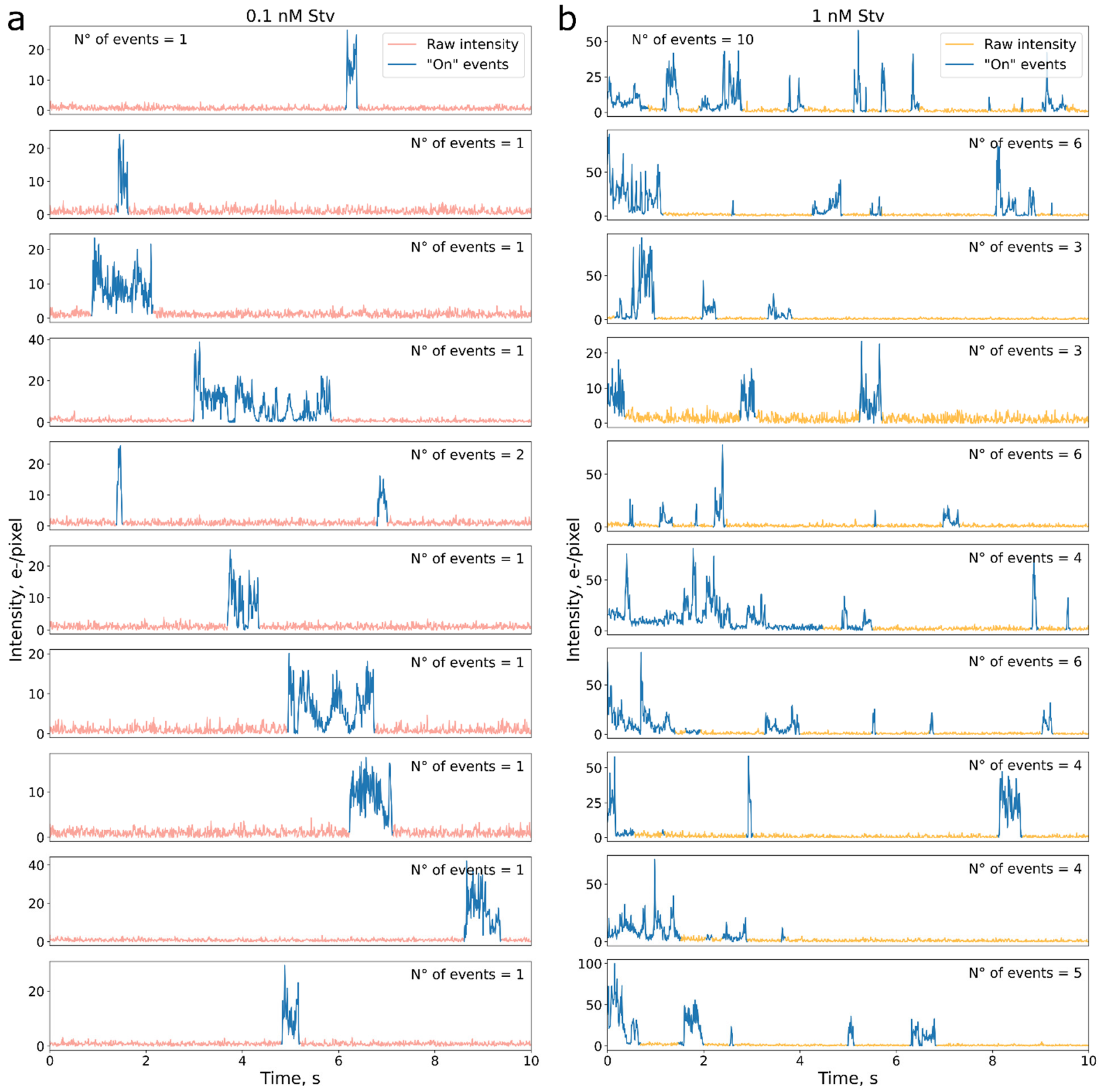

Figure S5. Representative examples of background-subtracted intensity evolution versus time for 10 individual NWs after incubation with (**a**) 0.1 nM (**b**) 1 nM A647-Stv. The intensity fluctuations were recorded for 10 seconds at a rate of 100 frames per second. The events with a certain intensity

above the background, determined by a user-defined threshold, are highlighted in blue, indicating the presence of at least one fluorophore diffusing on the surface of the inspected NW.

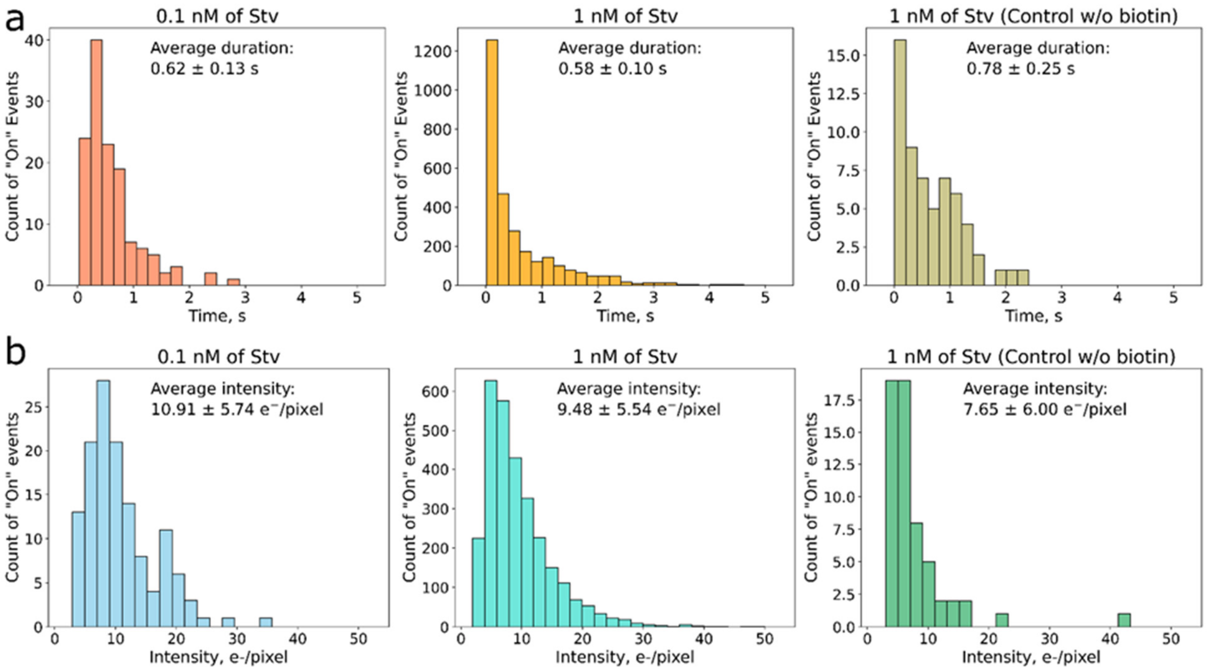

Figure S6. Distribution of (a) burst duration and (b) mean intensity from data shown in Figure S5, using all NWs in the field of view (9×9 μm²) for 0.1 nM (left panels) and 1 nM (middle panels) A647-Stv. The right panels include a control using 1 nM A647-Stv without biotin-modified lipids in the SLB. This data was extracted from intensity traces obtained from the coordinates of individual nanowires. The signals were convoluted with a Gaussian kernel (sigma = 2.5) to reduce noise, followed by identifying intensity bursts for signals above an intensity threshold of 3. Bursts separated by less than 0.2 seconds with detections below the threshold were combined into the same event. The similar duration (0.62 and 0.58 s) and mean intensities (10.9 and 9.5 e^{-}/pixel) of the bursts at 0.1 and 1 nM A647-Stv, respectively, are consistent with single protein detection, and the number of detections at 1 nM A647-Stv with biotin (~50 times higher than without biotin) verifies specific protein binding.

Section 7. Complementary movies.

The movies used to generate the data presented in this paper correspond to fluorescence microscopy micrographs and were acquired as described in sections 2.2 and 4.1:

Movie S1: FRAP 1_Fig 2a.avi. FRAP of the SLB shown in Movie 1/ Fig. S2a

Movie S2: FRAP 2_Fig 2b.avi. FRAP of the SLB shown in Movie 3/ Fig. S2b

Movie S3: SLB 1_Fig 2a.avi. SLB formation on Si NWs (data shown in Fig. 2a)

Movie S4: SLB 2_Fig 2b.avi. SLB formation on planar silica (data shown in Fig. 2b)

Movie S5: FRAP 3_Fig 3a.avi. FRAP of the SLB in Movie 3/ Fig. S3a

Movie S6: FRAP 4_Fig 3b.avi. FRAP of the SLB in Movie 4/ Fig. S3b

Movie S7: SLB 3_Fig 3a.avi. SLB formation on Si NWs (data shown in Fig. 3a)

Movie S8: SLB 4_Fig 3b.avi. SLB formation on planar silica (data shown in Fig. 5)

All the movies are in AVI format and saved at 5 frames per second.

They can be downloaded here: https://lu.box.com/s/7uwbdpv5nplskkp2x6vf5g5eg5n1m9v0 or by scanning the QR code:

REFERENCES

1. Thi Hoang Nguyen, V. *et al.* The CORE Sequence: A Nanoscale Fluorocarbon-Free Silicon Plasma Etch Process Based on SF $_6$ /O $_2$ Cycles with Excellent 3D Profile Control at Room Temperature. *ECS Journal of Solid State Science and Technology* **9**, 024002 (2020).

2. Philo, J. S. SEDNTERP: a calculation and database utility to aid interpretation of analytical ultracentrifugation and light scattering data. *Eur Biophys J* **52**, 233–266 (2023).

3. Rayan, G., Guet, J.-E., Taulier, N., Pincet, F. & Urbach, W. Recent Applications of Fluorescence Recovery after Photobleaching (FRAP) to Membrane Bio-Macromolecules. *Sensors* **10**, 5927–5948 (2010).

4. Reits, E. A. J. & Neefjes, J. J. From fixed to FRAP: measuring protein mobility and activity in living cells. *Nat Cell Biol* **3**, E145–E147 (2001).

5. Kohze, R., Dieteren, C. E. J., Koopman, W. J. H., Brock, R. & Schmidt, S. Frapbot: An open-source application for FRAP data. *Cytometry Part A* **91**, 810–814 (2017).

6. Koulouras, G. *et al.* EasyFRAP-web: a web-based tool for the analysis of fluorescence recovery after photobleaching data. *Nucleic Acids Res* **46**, W467–W472 (2018).

7. Transtrum, M. K., Machta, B. B. & Sethna, J. P. Why are Nonlinear Fits to Data so Challenging? *Phys Rev Lett* **104**, 060201 (2010).

8. Jönsson, P., Jonsson, M. P. & Höök, F. Sealing of Submicrometer Wells by a Shear-Driven Lipid Bilayer. *Nano Lett* **10**, 1900–1906 (2010).

9. Dabkowska, A. P. *et al.* Fluid and Highly Curved Model Membranes on Vertical Nanowire Arrays. *Nano Lett* **14**, 4286–4292 (2014).

10. Pincet, F. *et al.* FRAP to Characterize Molecular Diffusion and Interaction in Various Membrane Environments. *PLoS One* **11**, e0158457 (2016).

11. Wu, J., Shekhar, N., Lele, P. P. & Lele, T. P. FRAP Analysis: Accounting for Bleaching during Image Capture. *PLoS One* **7**, e42854 (2012).

12. Frederiksen, R. S. *et al.* Modulation of Fluorescence Signals from Biomolecules along Nanowires Due to Interaction of Light with Oriented Nanostructures. *Nano Lett* **15**, 176–181 (2015).

13. Macháň, R. & Hof, M. Lipid diffusion in planar membranes investigated by fluorescence correlation spectroscopy. *Biochimica et Biophysica Acta (BBA) - Biomembranes* **1798**, 1377–1391 (2010).

14. Andrecka, J., Spillane, K. M., Ortega-Arroyo, J. & Kukura, P. Direct Observation and Control of Supported Lipid Bilayer Formation with Interferometric Scattering Microscopy. *ACS Nano* **7**, 10662–10670 (2013).
